# DUX: One Transcription Factor Controls 2-Cell-like Fate

**DOI:** 10.3390/ijms23042067

**Published:** 2022-02-13

**Authors:** Wei Ren, Leilei Gao, Yaling Mou, Wen Deng, Jinlian Hua, Fan Yang

**Affiliations:** 1 College of Veterinary Medicine, Northwest A & F University, Xianyang 712100, China; r1159748661@163.com (W.R.); gaoleilei@nwafu.edu.cn (L.G.); 18523200300@163.com (Y.M.); jinlianhua@nwsuaf.edu.cn (J.H.); 2 Shaanxi Centre of Stem Cells Engineering & Technology, Northwest A & F University, Xianyang 712100, China; 3 College of Innovation and Experiment, Northwest A & F University, Xianyang 712100, China

**Keywords:** 2-cell-like cells, Dux, gene regulation, totipotency

## Abstract

The double homeobox *(Dux*) gene, encoding a double homeobox transcription factor, is one of the key drivers of totipotency in mice. Recent studies showed Dux was temporally expressed at the 2-cell stage and acted as a transcriptional activator during zygotic genome activation (ZGA) in embryos. A similar activation occurs in mouse embryonic stem cells, giving rise to 2-cell-like cells (2CLCs). Though the molecular mechanism underlying this expanded 2CLC potency caused by Dux activation has been partially revealed, the regulation mechanisms controlling Dux expression remain elusive. Here, we discuss the latest advancements in the multiple levels of regulation of Dux expression, as well as Dux function in 2CLCs transition, aiming to provide a theoretical framework for understanding the mechanisms that regulate totipotency.

## 1. Introduction

After fertilization, a zygote initiates a differentiation program contributing to all types of cells required by a new organism, owing to their “totipotent” developmental potency. As development progresses, cells derived from early embryos gradually lose their developmental potency; only the cells from 2-cell-stage embryos in mice and 4/8-cell-stage embryos from livestock can generate both embryonic and extra-embryonic cell types [1,2,3,4,5]. The widely used mouse embryonic stem cells derived from the E4.5 epiblast inner cell mass (ICM) are “pluripotent” owing to their ability to contribute to the somatic lineages and germline of the organisms [6]. Totipotent cells hold enormous potential for regenerative medicine. Thus, establishing a stable totipotent cell line is of paramount importance. However, no well-defined culture conditions have yet been established for the cells derived from zygotes and 2-cell embryos in vitro. The cells derived from early preimplantation embryos were reported to maintain self-renewal in long-term cultures and differentiate into all embryonic and extraembryonic cell lineages in mouse chimeras by using inhibitor cocktails to silence several signaling pathways [7]. However, these cells still express core pluripotency genes without specific defined totipotency markers, thus their developmental potency is controversial [8]. Even to date, bona fide totipotent embryonic stem cells have not yet been established, and our knowledge of totipotency is limited, partly due to the extremely limited cell number existing in early preimplantation embryos.

A transcriptome analysis of mouse preimplantation embryos revealed that the activation of unique transcripts takes place at the 2-cell stage but is undetectable at any other stages. These transcripts include, but are not limited to, zinc finger and SCAN domain containing 4 (Zscan4) [9,10,11,12,13,14], zinc finger protein 352 (Zfp352) [9], 2-cell-stage, variable group, member 1/3 (Tcstv1/3) [9], predicted gene 4340 (Gm4340) [11], TD and POZ domain containing 1–5 (Tdpoz1–5) [14] and procollagen-proline, 2-oxoglutarate 4-dioxygenase, alpha II polypeptide (P4ha2) [10], most of them able to generate chimaeric transcripts linked to murine endogenous retrovirus with leucine tRNA primer (MERVL) element. The MERVL-one class of endogenous retroviral elements (ERVs) and hundreds of genes driven by the 5′LTR of MERVL are upregulated specifically at the 2-cell stage. Meanwhile, 1% of a mouse ESCs (mESCs) population cultured in standard serum and LIF (SL) conditions expresses MERVL and a specialized gene set specific to the 2-cell stage [15]. These cells are named 2CLCs and have a developmental potency similar to their in vivo counterparts. However, our current understanding of the regulation of 2CLCs is largely limited to the identification and characterization of ESC-enriched coding genes that program the cell fate potential to a pluripotent state rather than activate it to the 2-cell-like state [16], and the key factors positively controlling totipotency need to be identified in 2CLCs. Recently, Dux was found to be activated in 2-cell embryos, and Dux overexpression can activate MERVL and 2-cell-specific transcripts, leading to the transition of pluripotent mESCs into totipotent 2CLCs. These studies have been summarized in a series of comprehensive review articles [17,18,19]. In this review, we focus on the regulation of the Dux gene transcript in 2CLCs and the functions of Dux in controlling cell fates. Finally, we connect the data of Dux expression in 2CLCs from different regulatory layers and discuss the existing knowledge gaps and prospective approaches to future studies of totipotent cells.

## 2. The Origin and Evolution of *Dux* Gene

In mice and rats, the intronless gene *Dux*, encoded by a 4.9 kb retrogene array of more than 28 copies, is located at chromosome 10, whereas 23 the *Dux* paralog copies in human double homeobox 4 (*DUX4*); only 1–12 bases different from mouse *Dux* have been identified, in which 14 encoding mRNAs are transcribed in 2-cell-stage embryos as *Dux* [20]. The further study of these 14 mRNAs’ expression regulation will help us to understand the difference between *Dux* and its paralog. The intronless orthologs to human *DUX4* are also found in other primates, elephants, hyraxes, and tenrecs. Similar to *Dux*, the *DUX4* family also contains a 3.3 kb copies array (D4Z4) located at the distal end of chromosome 4 [21]. An intron-containing variant, double homeobox B-like (*Duxbl*), is only found in rodents, and its pseudogene is found in primates [21]. The intronless *Dux*/*DUX4* was hypothesized to arise from the common intron-containing ancestor in placental mammals through the reverse-transcription and retrotransposition models [22]. During convergent evolution, environment pressure may further dictate the location of gene insertion. Genomic microsatellite organizations of *Dux* and *DUX4* are usually located at the heterochromatinized regions, and their expression is often silenced in most types of cells, including embryonic stem cells [9,23]. Epigenetic regulations, such as DNA methylation, are a key determinant for their expression [23]. Despite the considerable sequence divergence in their DNA-binding domains, DUX and DUX4 shared a more conserved homeodomain 2 (HD2) domain to recognize the 5′-TGA-3′ motif [24] and activate a subset of genes associated with cleavage-stage embryos [9]. Interestingly, the swapping of the homeodomain 1 (HD1) and HD2 regions of DUX4 with the corresponding regions from DUX substantially attenuates the activation of Zscan4c and MERVL induced by DUX expression in mouse muscle cells [25]. Additionally, mouse Dux is myotoxic and shares a partial functional homology with its human paralog DUX4, the aberrant expression of which is linked to facioscapulohumeral muscular dystrophy [26], which further confirms the conserved roles of Dux/DUX4 in gene regulation. 

## 3. The Regulation of Dux Expression

Dux mRNAs are expressed at the early 2-cell stage in mice during the minor zygotic genome activation (ZGA) stage, whereas it activates several genes during major ZGA, similar to DUX’s homolog DUX4 [20]. The overexpression of Dux in mESCs resulted in changes in gene expression and endowed these cells with totipotency [9,25,27]. Given its critical role in ZGA and the ESCs to 2CLCs transition, Dux expression must be tightly regulated to ensure the correct developmental progression of cells and tissues. Although transcriptome studies on mouse cells expressing Dux have been widely carried out in vivo and in vitro, the mechanisms for the regulation of *Dux* gene expression remain elusive. Thus, we will discuss the recent studies revealing the mechanisms modulating Dux expression, involving transcriptional regulation by transcription factors, epigenetic modifications, signaling pathways and 3D genome conformation.

### 3.1. Transcriptional Regulation of Dux Expression by Transcription Factors 

#### 3.1.1. DPPA2/4

**Figure 1:**
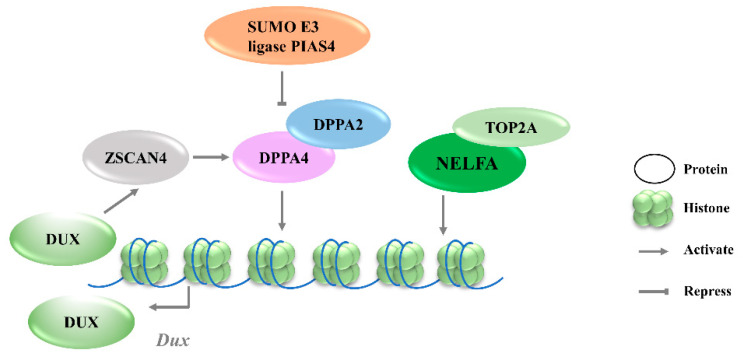
Transcriptional regulation of Dux expression by transcription factors. DPPA2/4 and NELFA transcriptionally activate Dux. Meanwhile, DUX can activate Zscan4 which will upregulate DPPA2/4. PIAS4 will repress DPPA2/4 expression through the SUMOylating of DPPA2. DPPA2/4, developmental pluripotency associated 2/4; PIAS4, protein inhibitor of activated STAT 4; NELFA, negative elongation factor complex member A; ZSCAN4, SCAN domain containing 4; TOP2A, DNA topoisomerase 2a; DUX, double homeobox.

Given that Dux is only expressed in the first or minor wave of ZGA, there must be other factors involved in the precise activation of its expression. By screening epigenetic factors that can increase 2CLCs population in normal ESCs, developmental pluripotency associated 2 (DPPA2) and developmental pluripotency associated 4 (DPPA4) have been identified as upstream factors of Dux that initiate 2C-like transcription [28]. DPPA2/4 are small putative DNA-binding proteins expressed exclusively in preimplantation embryos and pluripotent cells [29]. The overexpression of DPPA2/4 can activate an early 2-cell transcriptome, a similar pattern to that seen in mESCs. Additionally, ChIP-seq data reveal that DPPA2/4 can directly bind to the Dux repeats and the promoter region. However, DPPA2 or DPPA4 cannot work alone to activate Dux expression; these two factors must be present in equimolar amounts to transactivate Dux [30]. Recent studies further revealed that the DPPA2 activity is negatively regulated by a small ubiquitin-like modifier (SUMO) E3 ligasePIAS4, through the SUMOylation of DPPA2, which leads to its degradation (Figure 1). Either PIAS4 knockout or DPPA2/4 overexpression is sufficient to activate a 2C-like transcriptional program; the expressions of *MERVL* and other classic 2C-specific genes, including *Dux*, N-acetyltransferase 8 family member 2 (*Cml2*), *Zfp352*, and zinc finger and SCAN domain containing 4D (*Zscan4d*), are then upregulated [31].

#### 3.1.2. NELFA 

Negative elongation factor complex member A (NELFA) was another transcription factor reported to drive the progression to the 2CLCs state by activating Dux [32]. NELFA is a member of the NELF complex family that regulates RNA polymerase II pausing [33]. Unlike the enrichment of DPPA2/4 at the Dux locus, ChIP-seq data showed the low enrichment of NELFA at this locus [34]. However, upon NELFA induction, NELFA located at the Dux locus was responsible for the chromatin opening and the transcriptional activation of Dux. Specifically, the interaction of NELFA with DNA topoisomerase 2a (Top2a) is essential for NELFA to activate Dux; Dux will be silenced in Top2a-deficient cells even when NELFA is overexpressed (Figure 1) [32]. The role of NELFA remains controversial, as other studies indicate that NELFA is a direct target of DUX rather than a driver of Dux [34]. More data from Nelfa knock-out mESCs or embryos will help to clarify the role of NELFA in Dux regulation.

#### 3.1.3. ZSCAN4C

**Figure 2:**
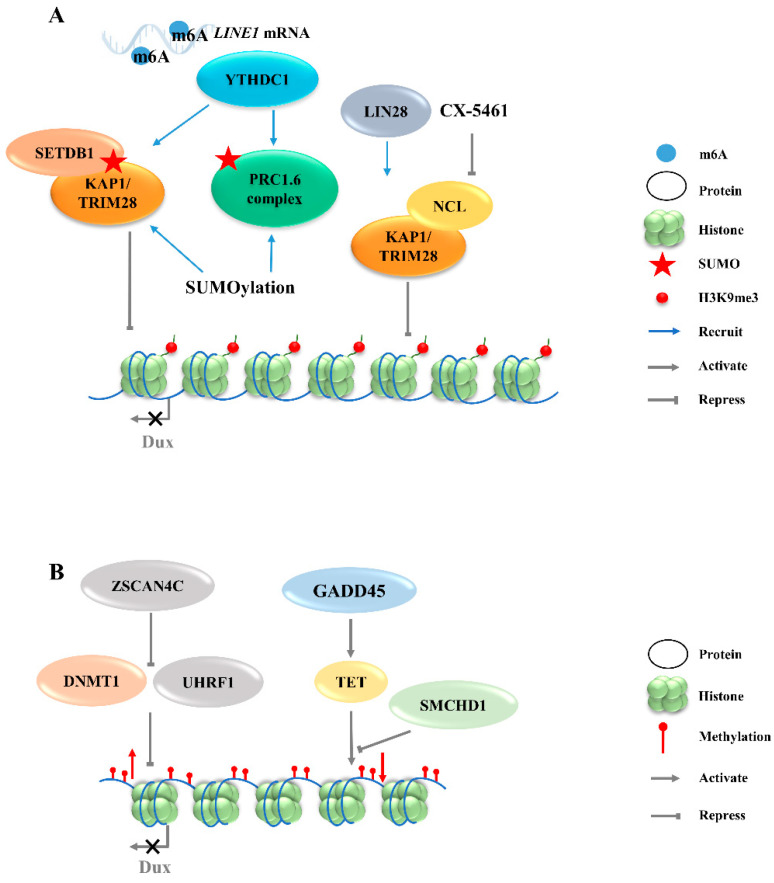
Regulation of Dux expression by epigenetic modifications. (**A**): Dux expression regulated by histone modifications transcription. The activation of H3K9me3 regulators caused by YTHDC1 and LIN28 will enhance the H3K9me3 levels and silence Dux expression finally. (**B**): Dux expression regulated by DNA modifications. DNA methylations caused by DNMT1/UHRF1 and TET repress Dux expression. LINE1, long interspersed nuclear element-1; SETDB1, SET domain bifurcated histone lysine methyltransferase 1; KAP1, KRAP-associated transcriptional repressor; TRIM28, tripartite motif-containing protein 28; PRC1.6, polycomb-repressive complexes 1.6; NCL, nucleolin; ZSCAN4C, SCAN domain containing 4C; DNMT1, DNA methyltransferase 1; UHRF1, ubiquitin like with PHD and ring finger domains 1; GADD45, growth arrest and DNA damage 45; TET, ten-eleven translocation; SMCHD1, structural maintenance of chromosomes flexible hinge domain containing 1.

Zinc finger and SCAN domain containing protein 4 C (ZSCAN4C), which shares a similar expression pattern to MERVL in normal mESCs, has also been identified as a 2CLCs marker [35]. The activation of MERVL by ZSCAN4C is associated with promoting enhancer activity and enhancing histone modification deposition related to gene activation at MERVL LTR loci [36]. Although Dux activation was observed after the overexpression of Zscan4c, the ChIP-seq data do not show the direct binding of ZSCAN4C in the Dux promoter region, suggesting an indirect transcriptional activation of Dux by ZSCAN4C binding [36]. However, ZSCAN4 has been demonstrated to facilitate gene expression by inducing global DNA demethylation through silencing the DNA methylation ubiquitin-like components, UHRF1and DNMT1, indicating an additional regulatory layer of Dux by ZSCAN4 (Figure 2A) [37]. Moreover, Dux regulators, including DPPA2 and DPPA4, are upregulated by ZSCAN4 overexpression, reflecting that ZSCAN4, DPPA2/4, and DUX may reinforce each other’s expressions and form a positive feedback loop to strengthen 2-cell-like state transition (Figure 1).

### 3.2. Regulation of Dux Expression by Epigenetic Modifications 

#### 3.2.1. H3K9 Methylation

The totipotent 2CLCs have also been reported to exhibit increased histone modifications in H3K27ac, H3K4me1, and H3K4me3, as compared with ESCs [38]. Although these histone modifications are associated with transcriptional activation, no evidence exists to show that Dux expression will be directly regulated by these modifications. The downregulations of chromatin modifiers such as LSD1 and chromatin assembly factor 1 (CAF-1) facilitate MERVL activation [15,39]. Furthermore, MERVL requires lysine (K)-specific demethylase 1A (KDM1A, also as LSD1)—a histone lysine-specific demethylase, a KRAP-associated transcriptional repressor (KAP1), and G9A—a H3K9 histone methyltransferase—for epigenetic repression in normal mESCs [27,40,41]. Likewise, there is no direct evidence showing that the expression of Dux can be regulated by these chromatin modifiers. Recently, LIN28, an RNA-binding protein, was identified as able to repress Dux by an epigenetic program (Figure 2A). H3K9me3 levels were decreased at Dux and its downstream targets, and thus de-repressed Dux expression in Lin28 knockout cells [11]. However, the mechanisms underlying how Lin28 regulates H3K9me3 remain elusive. It is worth noting that Lin28a depletion releases Dux repression by reducing the occupancy of Nucleolin/tripartite motif-containing protein 28 (NCL/TRIM28) in the Dux region. TRIM28 is also known as KAP1, which was demonstrated to repress Dux expression in a long interspersed nuclear element-1 (LINE1)-dependent manner in mESCs [27,42]. LINE1 are Class I transposable elements, which can repress Dux expression by interacting with NCL and KAP1 in mESCs [42]. Mechanistically, after LINE1 RNA is methylated by METTLE3, the m6A-modified LINE1 RNA then works as a scaffold recognized by the YTH domain containing 1 (YTHDC1), which further recruits H3K9me3 regulators, including SET domain bifurcated histone lysine methyltransferase 1 (SETDB1) and KAP1, to the locus of Dux, inhibiting its expression [43,44]. Furthermore, polycomb-repressive complexes (PRCs) bind LINE1 RNA and act as an essential partner for *Dux* gene repression [45]. SUMO modification enhances the H3K9me3 levels on a genome-wide scale, including the Dux locus, and facilitates the recruitment of PRC1.6 and KAP/SETDB1 complexes to the locus to repress *Dux* gene expression (Figure 2A) [46]. In fact, Ythdc1 depletion results in a global decrease in the SETDB1-mediated H3K9me3 enrichment, which is accompanied by the re-activation of MERVL and Dux [43]. However, Ythdc1-depleted cells still retain the ability to re-activate many retrotransposons upon Dux removal, indicating a parallel regulation pattern between Ythdc1 and Dux with regard to retrotransposon regulation [44]. Due to the sequence differences in LINE1 among species, it is not clear whether LINE1 has similar effects in other mammals, including *Homo sapiens* [47].

#### 3.2.2. Histone Variants

The chromatin assembly factor CAF-1 has been reported to repress MERVL [39], and recent studies revealed its role in establishing the modification of the non-canonical histone variant H3.3, which has been reported to co-enrich with H3K9me3 to silence ERVs in mESCs [48]. The knockout of p150, a subunit of the CAF-1 complex, leads to a decrease in the total H3.3 enrichment, accompanied by the upregulation of Dux and MERVL. ChIP-seq data further confirm that H3.3 is enriched at the *Dux* locus and represses Dux expression [49]. However, the incorporation of H3.3 chaperones HIRA, ATRX or DAXXis not necessary per se for the function of H3.3 in Dux repression, indicating the existence of other H3.3 chaperones that may regulate Dux expression [49]. 

#### 3.2.3. DNA Methylation

In addition to histone modification, there are additional epigenetic mechanisms that regulate the activity of Dux. Typically occurring at the cytosine in CpG, 5-Methylcytosine (5mC) is a critical modification in the development and differentiation of cell lineages by blocking gene transcription [50]. The structural maintenance of chromosomes flexible hinge domain containing 1 (SMCHD1) cooperates with ten-eleven translocation (TET) proteins to negatively regulate the activities in DNA demethylation (Figure 2B). The removal of SMCHD1 from mESCs induces TET-dependent demethylation, preferentially at SMCHD1 targeting sites, along with the activation of *Dux* and the *Dux* pseudogene (*Gm4981*) [12,51]. The siRNA-mediated knockdown of Smchd1 in zygotes leads to a continued overexpression of Dux through the 8-cell stage. In addition, the presence of an unmethylated state of the *Dux* promoter region in the 2-cell stage indicates that the initial activation of Dux DNA is demethylation-dependent [51,52]. However, the mechanism of re-methylation of the *Dux* locus occurring in the later stages of development remains elusive. Further studies are also needed to address whether SMCHD1 can inhibit TET proteins to modulate the DNA demethylation process. Growth arrest and DNA damage 45 (GADD45) is another regulator of TET-mediated DNA demethylation. The triple-knockout (TKO) of *Gadd45*a, b, and g in ESCs causes locus-specific DNA hypermethylation, along with the downregulation of 2C-specific genes, including *Dux*. The transient overexpression of Gadd45a, Gadd45b, or Gadd45g, individually or together, in a TKO background recovered Dux expression back to the control level [53]. These findings suggest that TET enzymes may play a dual role in regulating Dux expression when engaging with different partners. On the one hand, TET enzymes may play a repressive role when SMCHD1 represses the TET de-methylation function to silence Dux expression [12]. On the other hand, TET enzymes can play a promoting role in activating Dux expression through demethylation via physical interaction with GADD45 (Figure 2B). 

### 3.3. Signaling Pathways Involved in Regulation of Dux Expression

#### 3.3.1. Retinoic Acid Pathway 

**Figure 3:**
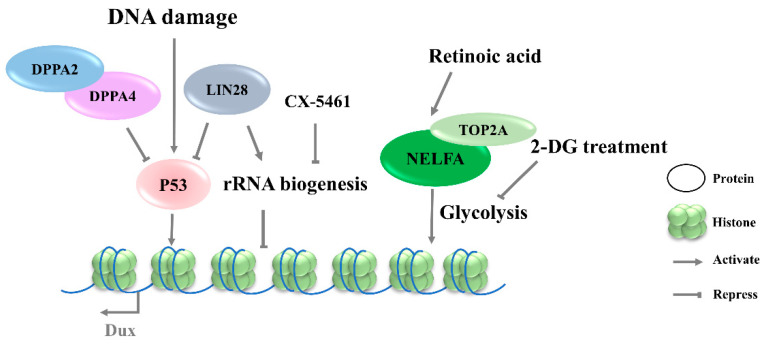
Signaling pathways involved in the regulation of Dux expression. DNA damage lead to P53 activation and then upregulates Dux expression. CX-5461 can decrease rRNA biogenesis and activate Dux expression. Retinoic acid participates in the NELFA-mediated Dux activation, and 2-DG inhibited glycolytic flux and activated Dux expression. DPPA2/4, developmental pluripotency associated 2/4; NELFA, negative elongation factor complex member a; TOP2A, DNA topoisomerase 2a; P53, tumor protein 53.

Retinoic acid (RA), a derived form of vitamin A (VitA), is involved in a variety of biological functions, including embryogenesis [54] and cell differentiation [55]. RA has been reported to initiate the reprogramming of ESCs to 2CLCs by co-activating Dux and Duxbl1, but the mechanism underlying Dux regulation by RA is not clear [56,57]. Iturbide et al. demonstrated that low concentrations of RA are sufficient to induce 2CLCs in cooperation with RARγ [58]. Interestingly, RA also participates in the NELFA-mediated 2C-like state of mESCs (Figure 3), but the downstream receptors have not been identified [56]. 

#### 3.3.2. Glycolysis Pathway

The 2CLCs exhibit decreased glycolytic and respiratory activity and lower levels of reactive oxygen species, but with an increased glucose uptake, suggesting a distinct metabolic state arising during the transition from ESCs to 2CLCs [59,60]. Regarding the mechanism underlying the NELFA control of Dux activation, the glycolysis pathway was found to be downregulated in NELFA-induced 2CLCs, along with a decreased chromatin accessibility of glycolysis-associated genes (Figure 3). Further investigation confirmed the inhibition of glycolytic flux by 2-deoxy-d-glucose (2-DG), which promoted the 2CLC state transition; naïve pluripotency stabilizer PR/SET domain 14 (Prdm14) serves as a barrier in this transition. The upregulation of 2C genes has been observed upon Prdm14 knockdown in mESCs [32,61]. However, it is unclear whether the alteration of the energy metabolism pathway is the driver of Dux activation, or if it is a consequence of Dux expression.

#### 3.3.3. DNA-Damage Response Pathway

Although DNA-damage-induced cellular differentiation was investigated in detail in ESCs [34,62], its role in totipotency regulation remains incompletely understood. Recent studies have shown that Dux activation in mESCs is initiated by the DNA-damage response (DDR) pathway, in which DNA damage-induced p53 activation plays an important role in regulating Dux expression (Figure 3). Double-strand DNA breaking induced by doxorubicin, PpoI endonuclease, hydroxyurea–aphidicolin [34], or UV [63] in mESCs results in higher levels of Dux activation and an increased MERVL-positive cell population. Consistent with these findings, p53 deficiency led to an almost complete loss of MERVL/Zscan4+ mESCs [63], accompanied by Dux repression [34]. ChIP-seq data on p53 further showed the direct binding of P53 in the Dux locus. In addition, Lin28 or Dppa2/4 deletion-mediated Dux activation is associated with p53 activation [11,28]. In contrast, in vivo data reveal that p53-knockout embryos are still positive for anti-DUX antibody staining, indicating that Dux activation is more complicated in in vivo settings [28]. The P53 regulation of mouse Dux and human DUX4 is conserved; however, the underlying molecular mechanisms are quite different. Although the intronless mouse Dux does not contain primate-specific LTR10C, which can be targeted by p53, p53 can bind to the Dux promoter directly to activate the transcription of mouse Dux [34]. 

#### 3.3.4. rRNA Biogenesis Pathway

**Figure 4:**
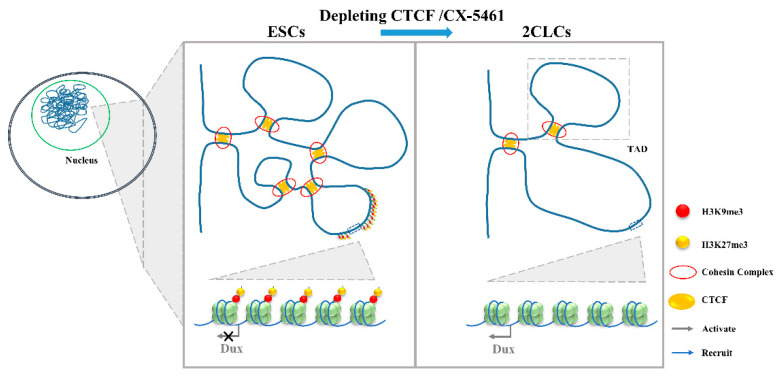
The 3D genome conformation determines the expression of Dux. The disruption of chromatin organization by the depletion of CTCF or addition with CX-5461 will lead to the lower TAD strength and TAD insulation and, finally, upregulate Dux expression. ESCs, embryonic stem cells; 2CLCs, 2-cell-like cells; CTCF, CCCTC-binding factor; TAD, topologically associating domains.

Protein synthesis heterogeneity requires the ribosome to be assembled in a cell-type-specific manner. Thus, differences in ribosome biogenesis can cause specialized mRNA subsets to be translated, which indirectly determine cell identity. Recent studies indicate that the inhibition of rRNA biogenesis can promote the state transition from ESCs to 2CLCs [11,13]. CX-5461, an RNA Pol I inhibitor, can decrease rRNA biogenesis and disrupt the nucleolar integrity and formation of peri-nucleolar heterochromatin (PNH). Upon the disruption of nucleolar integrity, the loss of NCL/TRIM28 complex from PNH causes changes in epigenetic modification, leading to the release of Dux (Figure 4) [13]. Transcription factor LIN28 has been reported to bind to RNAs in the nucleolus, and its loss impeded ribosome assembly and recapitulated CX-5461-induced 2CLCs molecular phenotypes [11]. In addition, rRNA repression will activate p53, an effector of DNA-damage response pathway [34]. Nevertheless, the connection to the mechanism of Dux production by transcriptional regulation and specific pathways observed in Dux activation remains to be elucidated. Meanwhile, how the different signaling pathways cooperate during Dux activation requires further elucidation.

### 3.4. 3D Genome Conformation

Gene expression regulation is relevant to chromatin structure [64], and Hi-C studies profiling the 3D chromatin structure of the genome could describe the evolutionarily conserved topologically associating domains (TADs) that were correlated with gene expression. Combined with mouse and human models, TADs have been linked to gene regulation. During the mESC to 2CLC transition, a lower TAD strength and TAD insulation have been observed [39]. Consistent with this is the disruption of chromatin organization by the depletion of CCCTC-binding factor (CTCF)/Cohesin or by culturing with CX-5461 upregulate Dux expression and promote a 2-cell-like program [13,14]. It is worth noting that the attenuation of H3K9me3 and H3K27me3 in CX-5461-treated cells is associated with the reinforcement of a repressed chromatin state [65]. The expression of human DUX4, the C-terminal region, which shares a high similarity with mouse DUX, can recruit CBP and P300 to induce local chromatin relaxation, accompanied with a global increase in H3K27 acetylation [66]. These results indicate that epigenetic modification is important for the chromatin state change [67]. Chromatin relaxation facilitates the binding of transcription factors to the Dux locus, which might be the first step required to promote Dux expression [14,39]. The expression of DUX increases the active histone modifications and chromatin accessibility [9], and this positive feedback loop might be required to promote the 2C-like state (Figure 4).

## 4. Functions of Dux in Cell Fate Transitions

DUX overexpression leads to the reprogramming of ESCs into a 2CLCs state through the direct transcriptional activation of 2-cell-related genes. Meanwhile, our previous studies also established an indirect role of Dux in 2CLCs reprogramming via a Dux–miR-344–Zmym2/Lsd1 axis [10]. It is worth mentioning that the MYM-type zinc finger domain of zinc finger MYM-type containing 2 (ZMYM2) contains SUMOylating sites, which were suggested to be SUMOylated by PIAS4, and represses the 2CLCs transition [31,68]. Dux–Klf5 is another axis controlling MERVL activation for expanded stem cell potency. Kruppel-like factor 5 (Klf5) is strictly regulated by Dux, and Klf5 overexpression can specifically upregulate *MERVL* and other 2-cell-associated genes [69]. In ESCs, although the ZGA transcriptional pattern caused by Dux overexpression has been extensively studied, there are currently no in vivo data to further confirm whether DUX overexpression cells can indeed contribute to extraembryonic tissues after injection into early embryos. Stringent criteria should be applied to evaluate the 2CLCs induced by Dux overexpression. Meanwhile, Dux only has minor effects on ZGA in vivo, since with the zygotic depletion of Dux, the embryos can still survive to adulthood in vivo [70,71,72]. However, the activation of some ZGA transcripts is delayed in Dux KO embryos, and mating pairs generate less offspring than wild-type controls [73]. These findings strongly indicate that Dux is important, but not essential, for in vivo embryo development. It is possible that a redundancy exists to regulate the successful totipotency of 2-cell embryos.

Nuclear transfer is another method by which mature cells can obtain totipotency. Dux expression was reported to improve the efficiency of somatic cell nuclear transfer (SCNT) [72,74]. Moreover, the transcriptome profiling of these Dux-treated SCNT embryos revealed their similarities to fertilized embryos. Mechanistically, Dux expression can rescue aberrant H3K9ac acetylation in SCNT embryos, accompanied by local chromatin relaxation, the activation of 2-cell embryo-related genes, and improved SCNT efficiency [74]. Further studies are required to reveal the epigenetic regulators involved in DUX-dependent H3K9ac activation.

## 5. Problems and Future Perspectives

Strictly speaking, totipotency refers to the ability of a single cell to form an entire organism, as well as extraembryonic tissues. As current totipotent cell models were established in vitro only, our understanding of totipotency is limited. Analyzing 2CLCs is important to our understanding of the mechanism of omnipotence in culture. A transcriptome analysis revealed an intermediate state existing in 2CLCs induced by Dux overexpression. During this intermediate state, the expression levels of pluripotency genes gradually decrease, whereas the expression levels of 2-cell-specific genes and MERVL increase [75]. The 2CLCs established in vitro are often associated with heterogeneity, and even Dux overexpression can only achieve a ~50% MERVL-positive cell population. Meanwhile, Dux is not essential for in vivo embryo development, which is the fundamental difference between the in vitro 2CLCs state and 2-cell embryos [70,71]. This indicates the existence of compensation mechanisms that likely trigger and maintain totipotency in vivo. It should be noted that nearly all of the currently established 2CLCs rely on gene-editing technology, which may impede the application of 2CLCs for regenerative medicine purposes. The identification of small molecules that promote *Dux* or MERVL expression could be of benefit to gene-editing-free 2CLCs and early embryo cultures in vitro.

Aside from 2CLCs, three more cell lines, reported to possess expanded potency, have been established in vitro. (1) Totipotent blastomere-like cells (TBLCs): spliceosome activation drives the totipotent to pluripotent stem cell transition during human and mouse embryo development. The culture of mESCs with pladienolide B, a splicing inhibitor, allows the conversion of mESCs to a totipotent state in vitro [76]. However, whether this culture condition can be applied to other species, such as human ESCs, remains unknown. (2) Expanded potential stem cells (EPSCs): inhibitors with the ability to modulate pluripotency gene expression and trophectoderm/ICM segregation have been screened and used to stabilize the naive pluripotency state. Several specific combinations of these inhibitors that appeared to achieve this purpose for mouse or human cells were identified. Chimera assays of these newly obtained cells revealed an expanded developmental potential, suggesting that they can be incorporated into extraembryonic tissues [7,77]. However, a transcriptome analysis identified the co-expression of pluripotency markers in these cells, alongside extraembryonic endoderm markers. In addition, their expanded development potential has been challenged, and no detectable contribution of EPSCs towards the TE lineage and blastoid formation was achieved when blastoids were generated by the combination of ESCs with trophoblast stem cells [78,79]. Compared with blastoid assay, chimeras generated by EPSCs indicated that the progeny of EPSCs localize to trophectodermal positions, but these cells do not express Sox2, a key TE marker [8]. (3) Hex+ cells: Hex is an extraembryonic endoderm marker, expressed spontaneously in a small population of naïve mESCs. Hex-reporter-positive cells can contribute to both embryonic and extraembryonic lineages [80]. However, stable Hex+ cell lines have yet to be established in vitro. Taken together, to date, no bona fide totipotent embryonic stem cells have been established in vitro. Hence, we propose that, to truly uncover the developmental potential of a cell into all extraembryonic and embryonic lineages, cells with expanded developmental potential should be sorted and analyzed by single-cell sequencing, and totipotency markers should also be determined as an initial step. Recent advances in sequencing technologies make it possible to establish the golden markers of totipotency. Cells from 2CLCs, TBLCs, and EPSCs should be analyzed with early embryos at the single-cell level. Cells with a similar transcriptome to 2-cell embryos can be sorted to further detect common regulators or pathways in totipotency regulation. Meanwhile, future work is urgently needed to reveal the comprehensive mechanisms of Dux regulation, including those of protein interaction and epigenetic regulation. In addition, since the expanded development potential of cells expressing Dux regulators has been identified by different groups, relying on a variety of assays of variable stringency, these cells varied in terms of genetic background, culture conditions and methods of MERVL-reporter insertion. Stringent criteria should be applied to estimate totipotency, as well as candidates for Dux regulation in mice.
